# Expression of insulin pathway genes during the period of caste determination in the honey bee, *Apis mellifera*

**DOI:** 10.1111/j.1365-2583.2006.00681.x

**Published:** 2006-10

**Authors:** D E Wheeler, N Buck, J D Evans

**Affiliations:** * Department of Entomology, University of Arizona Tucson AZ USA; † USDA ARS, Bee Research Laboratory Beltsville, MD 20705 USA

**Keywords:** caste determination, insulin-like peptide, insulin receptor, Chico, PTEN

## Abstract

Female honeybees have two castes, queens and workers. Developmental fate is determined by larval diet. Coding sequences made available through the Honey Bee Genome Sequencing Consortium allow for a pathway-based approach to understanding caste determination. We examined the expression of several genes of the insulin signalling pathway, which is central to regulation of growth based on nutrition. We found one insulin-like peptide expressed at very high levels in queen but not worker larvae. Also, the gene for an insulin receptor was expressed at higher levels in queen larvae during the 2nd larval instar. These results demonstrate that the insulin pathway is a compelling candidate for pursing the relationship between diet and downstream signals involved in caste determination and differentiation.

## Introduction

Reproductive division of labour, in the sense that one or a few colony members reproduce at a high rate while others do not, is a defining trait of highly social insect species (Wilson, 1971). This division is generally set during the larval stage when individuals can alter development sufficiently to produce different adult castes with distinctly different reproductive capabilities. The fact that caste determination of queens and workers in honey bees is effected through differential nutrition has been known for nearly 200 years (Huber, 1821). Adult honey bees have precise control over the caste of female offspring, and use this control to raise queens in response to loss or poor performance of the existing queen and in preparation for colony division. Queens are raised in developmental chambers (cells) that are enlarged and reoriented during development. Potential queens are fed the secreted substance royal jelly throughout development, and it is evident that both the quantity and quality of this diet are key proximate factors in initiating and maintaining a queen developmental path. Beekeepers have long exploited the social cues important for queen production, by first removing any existing queens and then offering colonies developmentally labile (first instar) larvae in wax cells that are orientated so as to mimic natural queen cells (Laidlaw, 1992; Michener, 1974). While the proximate cause of the queen-worker developmental switch in honey bees has been known for some time, the physiological mechanisms behind this switch remain elusive. Recent efforts to monitor gene-expression changes in female larvae as they approach and pass the developmental point of caste determination have suggested mechanistic routes for this switch (e.g. (Evans & Wheeler, 2000; Hepperle & Hartfelder, 2001)) and have begun to place this switch into the general context of developmental plasticity (Evans & Wheeler, 2001).

A central player in relaying nutritional information in a wide variety of organisms is the insulin signalling pathway (Ikeya *et al*., 2002; Nijhout, 2003). This pathway is an evolutionarily conserved module that acts as a rheostat for regulating growth and size (Oldham *et al*., 2002). The presence of an insulin-like peptide in honey bees was first suggested by immunoreactivity of head extracts to antibodies made to porcine insulin. These extracts also stimulated insulin-like activity in rat adipocytes and displaced porcine insulin from insulin receptors (O’Connor & Baxter, 1985).

**Figure 1:**
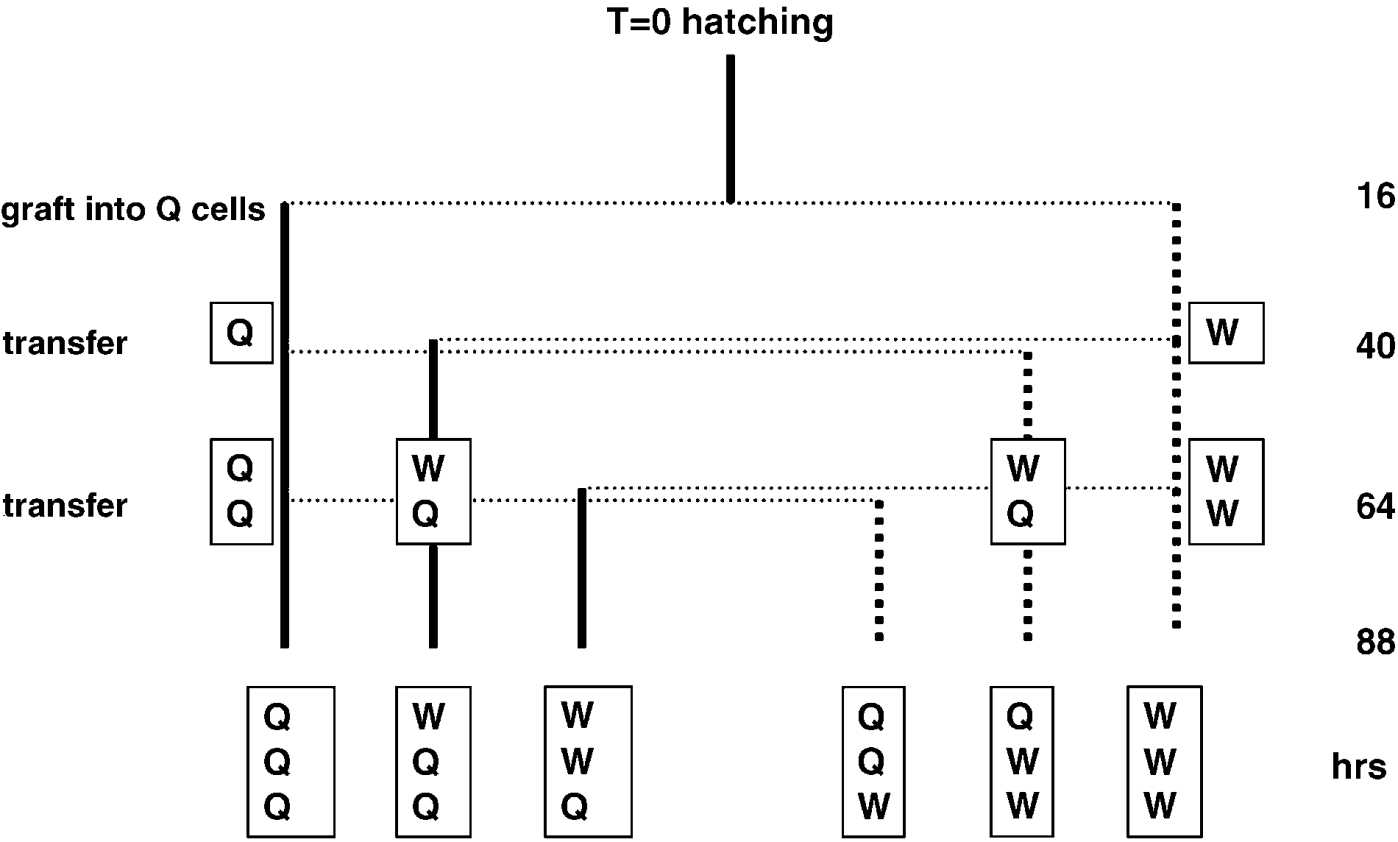
Experimental design for manipulation of larval diets. Larvae 16 hrs post hatching were either grafted into queen cells or remained in worker cells. At 40 and 64 hours, larvae that had spent 1 or 2 days in queen or worker cells, respectively, were transferred to the alternate food type. The design yielded 12 treatments that varied by age and diet history. QQQ and WWW are normally developing queen and workers.

**Figure 2:**
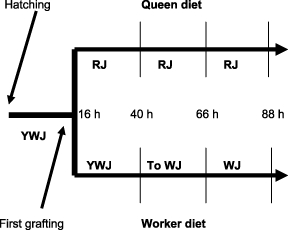
Diets by day for female larval bees in worker and queens cells. Nurse workers produce different larval diets through different mixtures of glandular secretions, crop contents and, in older worker larvae, pollen. A white component is produced by the mandibular glands and a clear component is a mixture of hypopharyngeal gland secretions and crop contents. The royal jelly (RJ) placed in queen cells is made up of equal amounts of the white and clear components. Nurse workers provision developing workers during their first 2 days with a mixture of 20–40% white secretion and the remainder the clear substance. Therefore, young worker jelly (YWJ) can be nearly as rich as royal jelly. On the third day, worker food becomes almost entirely clear and pollen is added, making worker jelly (WJ) (Jung-Hoffman, 1966 in Michener, 1974).

Here we begin to test the hypothesis that insulin plays a role in caste determination in honey bees. Using qPCR, we examined expression of genes encoding members of the insulin pathway. We focused on the early period in larval development when developmental pathways start to diverge, that is during the 2nd, 3rd, and 4th larval instars (40–88 h after hatching). We used reciprocal transfers of larvae between queen- and worker-driving environments (Fig. 1) to discover the proximate changes in expression of components of the insulin signalling pathway that occur in response to changes in diet quality (Fig. 2). This approach has been used historically to narrow the window for which caste changes are possible (Weaver, 1957).

## Results

### Am insulin-like peptide 1 (AmILP-1)

**Figure 3:**
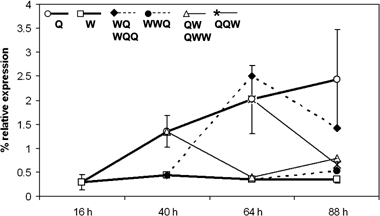
Expression of *AmILP-1*, an insulin-like peptide that is highly expressed in queen larvae. Standard errors are shown only for queen and worker larvae for ease of viewing.

*AmILP-1* mRNA expression varied dramatically with treatment (Fig. 3). Worker expression was consistently low while expression in queens increased over time. Queen levels were already high just 24 h after grafting (mid-2nd instar, see methods). When worker larvae were transferred to queen cells at the 2nd instar (40 h) instead of at the typical grafting age of 16 h, expression of *AMILP-1* rose sharply up to levels of queen larvae. When larvae were grafted from worker to queen cells at 60 h (3rd instar), expression levels did not increase above those of larvae that had remained in worker cells. When transfers were made in the opposite direction at both 40 and 64 h, from queen to worker cells, expression dropped to worker levels.

### Am insulin-like peptide 1 (AmILP-2)

**Figure 4:**
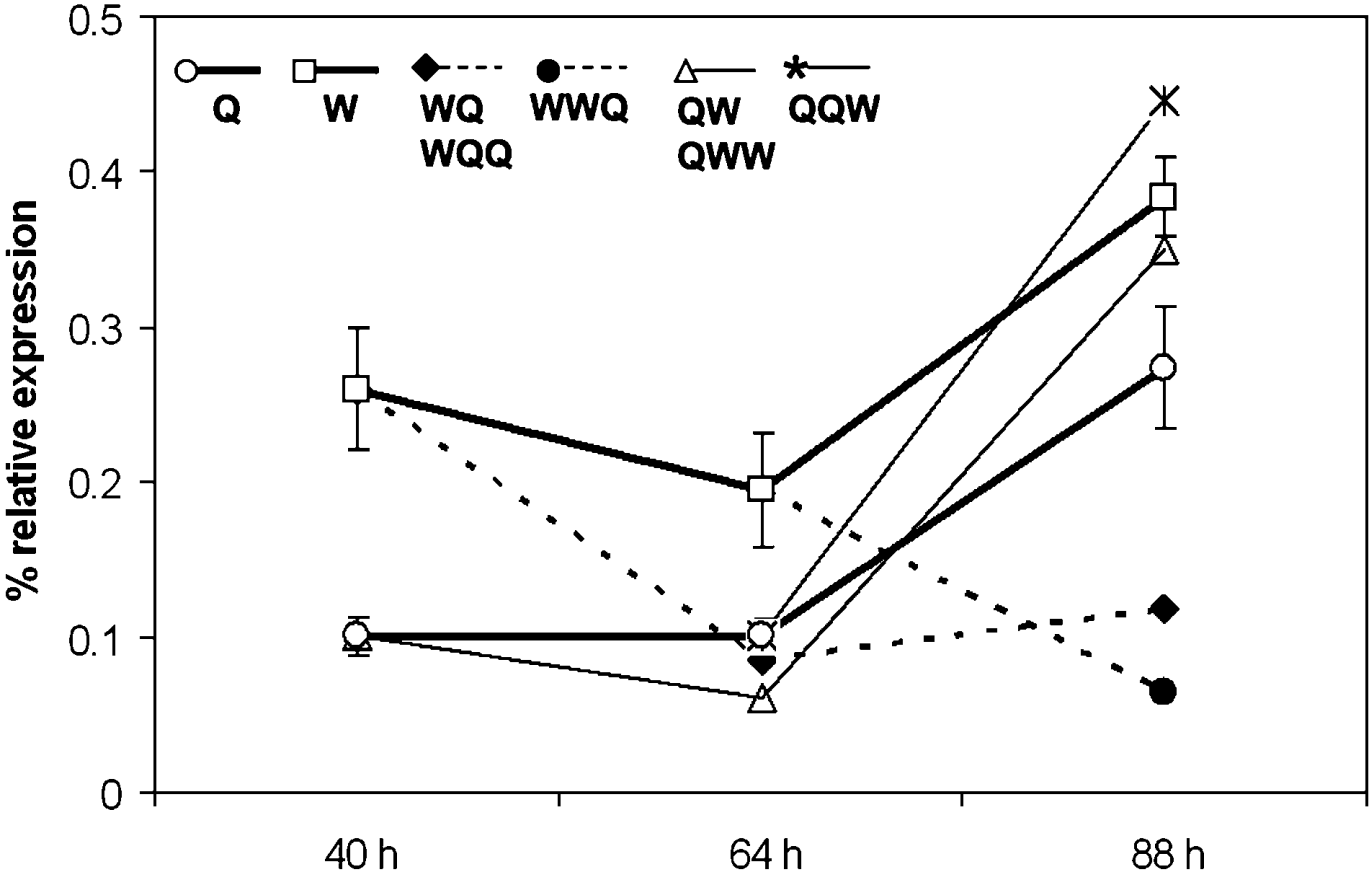
Expression of *AmILP-2*, a second insulin-like peptide. Standard errors are shown only for queen and worker larvae for ease of viewing.

Levels of *AmILP-2* were generally higher in workers than in queens (Fig. 4). For both castes, levels rose sharply during their fourth day. Transferring larvae from worker to queen cells caused expression to drop to queen levels, and transferring larvae from queen to worker cells caused expression to increase up to worker levels by the 4th instar.

### Insulin Receptor (AmIR-2)

**Figure 5:**
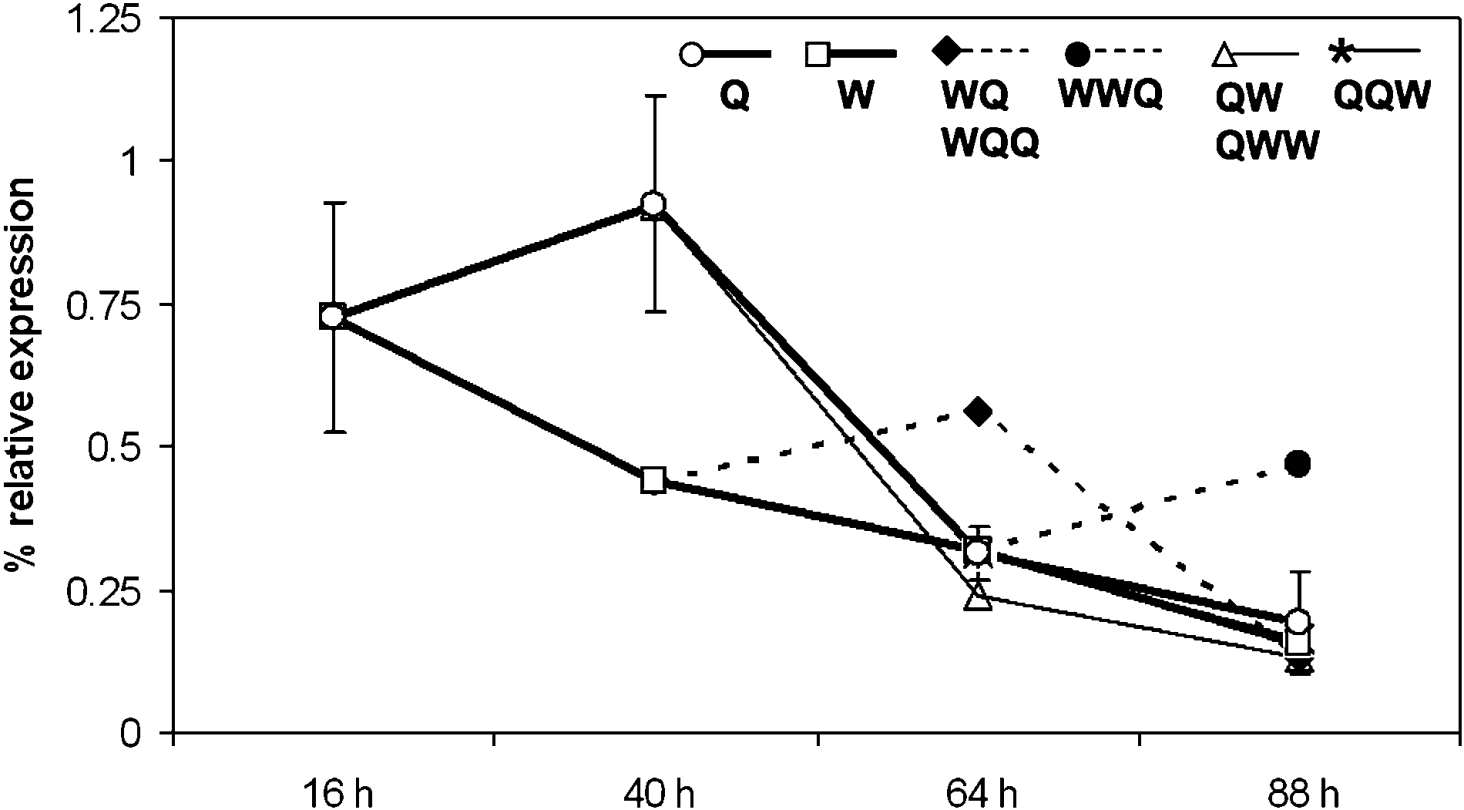
Expression of an Am insulin/IGF receptor protein, *AmInR-2*. Expression is high in young grafting age larvae and reaches even higher levels in queen-destined larvae at 40 hours. Standard errors are shown only for queen and worker larvae for ease of viewing.

Expression of insulin receptor gene *AmIR-2* was high in undifferentiated first instar larvae, and reached an even higher level in queen larvae during the 2nd instar (Fig. 5). Worker levels were consistently low. By the 3rd instar (64 h) expression in normal queens and workers were low and similar. Switching larvae from worker to queen cells at any time (Q, WQ, and WWQ) provoked a suggestive increase in receptor expression.

### Insulin Receptor Substrate (AmIRS, Chico)

**Figure 6:**
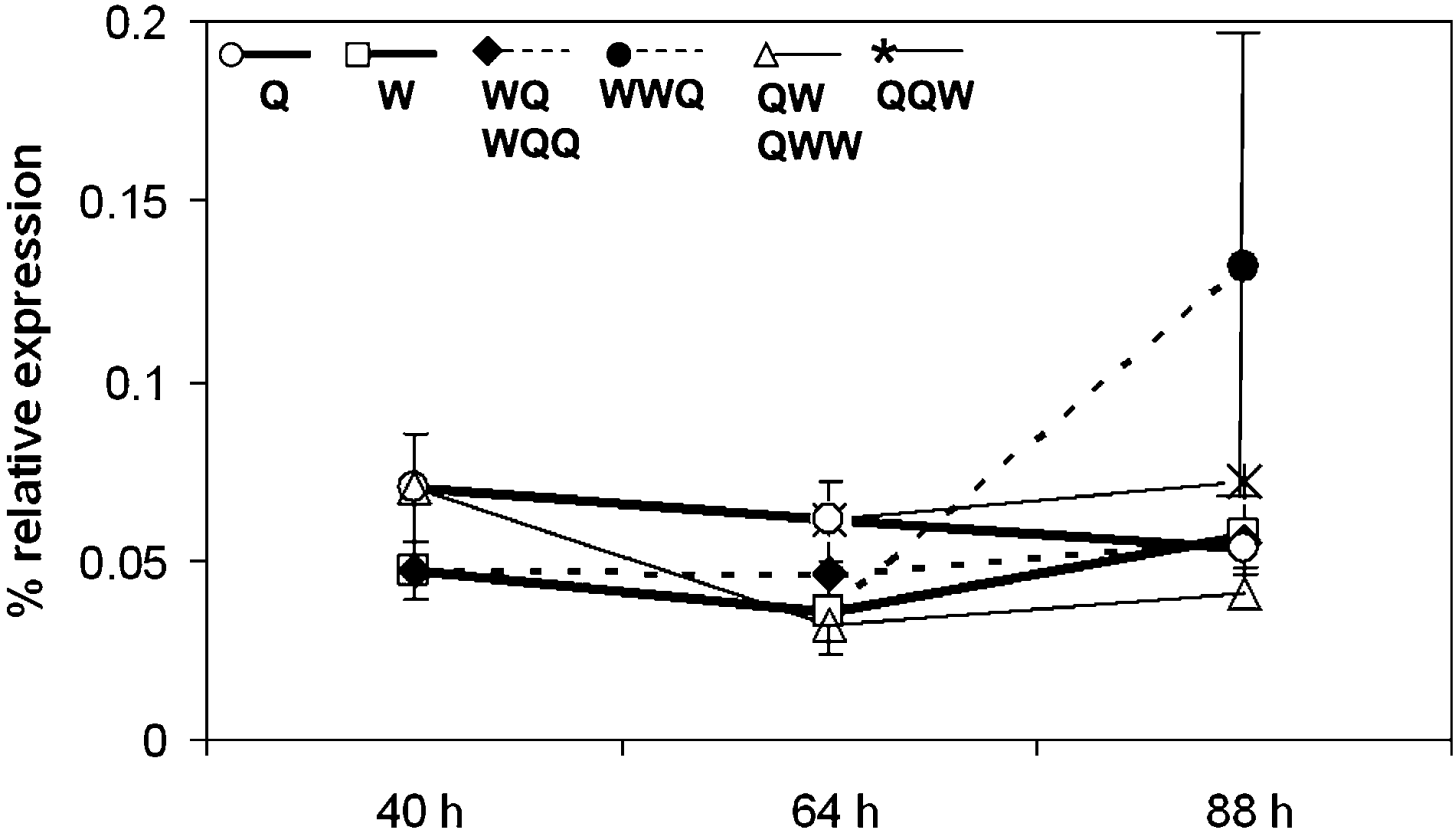
Expression of Am insulin receptor substrate, *AmIRS*. Differences among treatments were not significant. Standard errors are shown for WWQ, which showed a statistically non-significant increase, in addition to those for queen and worker larvae.

Expression of insulin receptor substrate was low and similar in both queen and worker larvae with no overall significant differences between treatments (anova, *P* > 0.155) (Fig. 6). When worker larvae were switched to queen cells at 48 h postgrafting (WWQ), expression appeared to rise sharply, but the level was not significantly different than that of WWWs.

### PTEN (AmPTEN)

**Figure 7:**
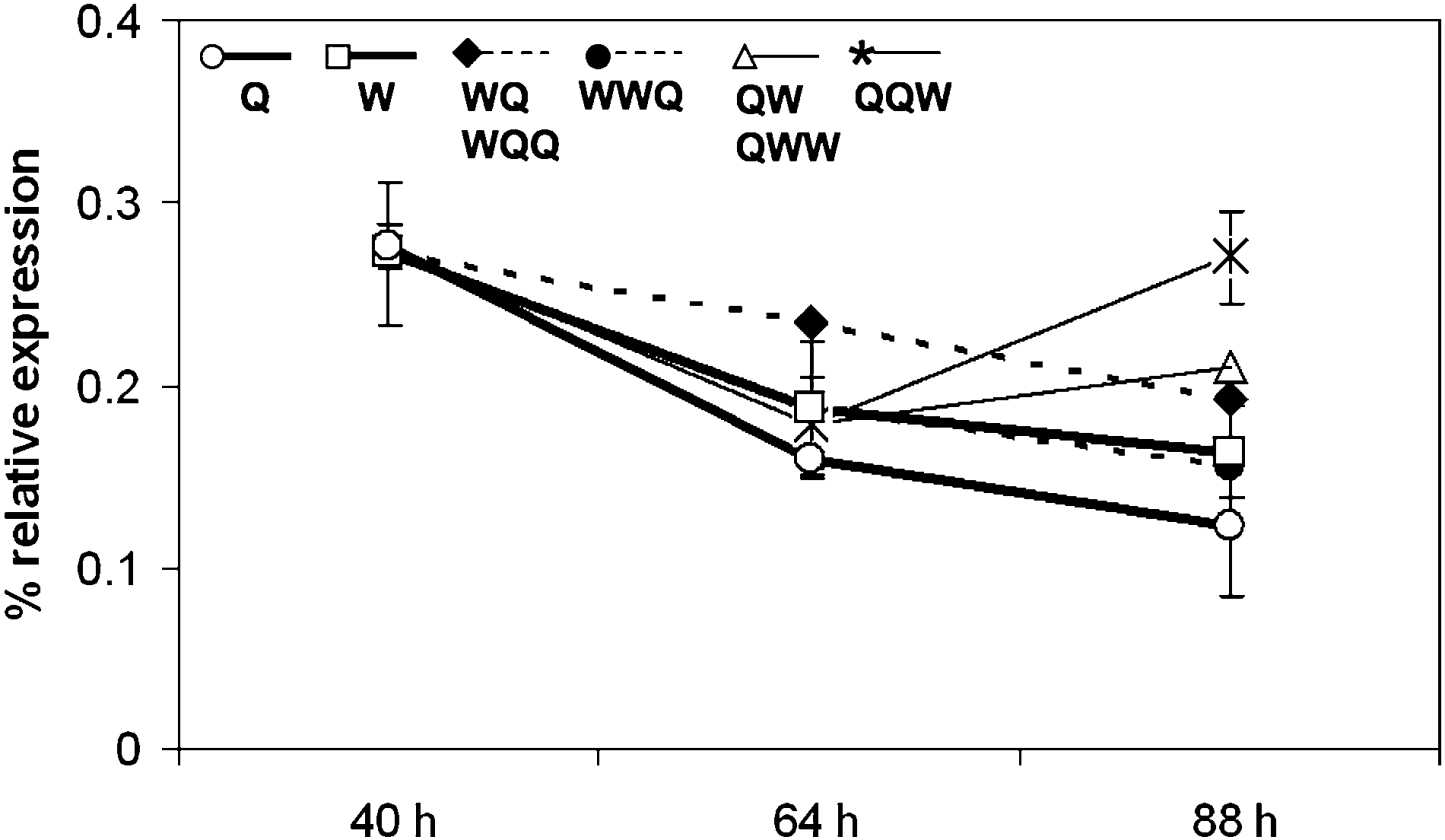
Expression levels of *AmPTEN.* Expression in queen and worker larvae was the same. Only the transfer of queen larvae to worker cells during the 3rd instar caused a significant change in expression. Standard errors are shown for QQW, which showed a statistically significant increase, in addition to those for queen and worker larvae.

Expression levels of *AmPTEN* were relatively low in both workers and queens and fell slightly over time (Fig. 7). The only manipulation that provoked a significant change from the slight downward trend with age was the increase in expression following transfer of queen larvae to worker cells as 3rd instars (*P* < 0.05).

## Discussion

Caste determination in honey bees is a classic case of nutritional determination of alternative phenotypes: queens and workers. Based on the information that insulin signalling is important in integrating nutrition and metabolism in a wide variety of organisms (Nijhout, 2003), we investigated expression of genes encoding five proteins in the insulin signalling pathway to determine if they were differentially regulated during development of workers and queens. *AmILP-1* and *AmILP-2* are small insulin-like peptides while *AmIR-2*, *AmIRS* and *AmPTEN* are, by homology, larger proteins that are part of the insulin signalling cascade that respond to the ILPs. We focused our investigation on the period in which nutritional and hormonal differences can affect caste development, during the 2nd, 3rd, and 4th instars (40–88 h in larval age). *AmILP-1*, *AmILP-2* and *AmIR-2* showed differences in expression between normally developing workers and queens as well as in response to shifts in diet. *AmPTEN* showed a change in response to diet, and *AmIRS* showed no significant change with either age or diet.

### Expression patterns

#### AmILP-1

By far the most highly expressed of the genes studied was *AmILP-1*. Expression in queen larvae was at levels well above that of the reference gene and 3–5 times higher than that of workers. When worker larvae began receiving royal jelly late, as 2nd instars (24 h postgraft), they increased *AmILP-1* expression to match or exceed queen levels by the 3rd instar. Third instar larvae, however, did not respond to being transferred to the richer food, suggesting that royal jelly induces up-regulation of this gene during a defined window that has closed by the 3rd instar. The inability of older larval bees to up-regulate *AmILP-1* when moved to a queen environment, coupled with the predicted roles of this protein during the insulin cascade, make this a compelling gene for functional studies of caste mechanisms. Under natural conditions, larvae can be exposed to food switches going from W to Q conditions after the death of their queen. The colony has at most 5 days, 3 for egg development and 2 for larval development up to 48 h, to construct queen cells around eggs or larvae and successfully produce a replacement queen. In contrast, switches from royal to worker jelly would not be encountered naturally. Switching queen larvae to worker food as 2nd or 3rd instars caused a drop in expression down to worker levels suggesting that royal jelly is needed to sustain *AmILP-1* expression.

#### AmILP-2

Expression of *AmILP-2* was higher in workers than queens from the 2nd instar onward. The level increased in both castes as the larvae began to grow more rapidly. Surprisingly, queen larvae are slightly smaller than workers until the beginning of the 4th instar ((Shuel & Dixon, 1968) and our observations). The relative size of queen and worker larvae is reversed as larvae pass about 80 mg in weight and worker larvae eventually reach a maximum of about 160 mg while queens are twice that, about 320 mg (Stabe, 1930; Wang, 1965). The patterns of expression of normally developing queens and workers indicate that expression level is correlated with growth rate rather than caste, a pattern that suggests *AmILP-2* is an insulin-like growth factor. Switching worker larvae to queen cells at either the 2nd or 3rd instar caused a reduction in expression down to levels near that of queens. Similarly, switching queen larvae to worker cells at these developmental ages provoked an increase in expression at 88 h, up to the level seen in similarly aged workers.

#### AmIR-2

Insulin receptors, along with *AmIRS* and *AmPTEN*, are part of the machinery of the insulin signalling pathway. This machinery would be expected to be in place early in all larvae since any of them could be reared in either cell type. In support of this expectation, *AmIR-2* expression was low in both queen and worker larvae during the 3rd and 4th instars in all treatments. Counter to this expectation, expression was at its highest in 2nd-instar queen-destined larvae, suggesting that an increase in receptor number is one of the first changes in expression in response to an extended diet of royal jelly. Increases in expression of *AmIR-2* also occurred after later transfers to Q cells (WQ, WQQ).

#### AmIRS and AmPTEN

Expression levels of *AmIRS* and *AmPTEN* fulfil the expectation that the proteins in the insulin signalling pathway are expressed at a low level to sustain replacement. They were expressed at low levels throughout the experimental period. The only deviation from this pattern was for *AmPTEN* in queen larvae switched to the worker diet as 3rd instars. PTEN is a phosphatase that inhibits the insulin pathway and down regulation of the IR/PI3K pathway is essential for responding appropriately to inferior nutrition (Britton *et al*., 2002). QQW larvae, for which royal jelly was replaced with the inferior worker jelly in the 3rd instar, expression rose sharply, suggesting that sudden deterioration in food quality causes enhanced inhibition of the response to an insulin-like peptide.

### Potential relationship of insulin signalling to juvenile hormone synthesis

The role of juvenile hormone (JH) in caste determination in honey bees is well known, with differences in larval diet correlated with differences in hormone titres (Rachinsky *et al*., 1990). Nurse workers produce different larval diets through different mixtures of glandular secretions, crop contents and, in older worker larvae, pollen. Morphological changes in queen and worker larvae appear during the third day and instar when the size of the corpora allata of queen larvae increases more quickly than those of workers (Dogra *et al*., 1977). Then, in queen larvae, both JH synthesis by the corpora allata *in vitro* and JH titres peak during the fourth day and instar *in vivo* (Rachinsky *et al*., 1990; Rembold *et al*., 1992). At this time queen larvae are also the most sensitive to treatment by exogenous JH (Wirtz, 1973). Switching diets at this time is no longer effective in producing functional queens. The system can be pushed, however, by switching larvae to royal jelly and treating them with JH (Dietz *et al*., 1979). The combination probably mimics an earlier switch to royal jelly, by adding supplementary JH to the small amount that can be stimulated endogenously by the late transfer.

In honey bee queens, both *AmILP-1* and *AmIR-2* are expressed at high levels by 24 h post grafting (40 h in age). JH levels begin to rise towards the end of this period, peak during the 3rd instar (∼48–72 h), and remain high for the duration of the larval period (Rembold *et al*., 1992). The temporal relationship of diet, levels of *AmILP-1* and *AmIR-2*, and JH titre differences is extremely suggestive, but non-etheless circumstantial. In many insect species, nutrition and JH levels are correlated in adult females, with better nutrition resulting in higher JH levels and, as a result, egg production (Wheeler, 1996). But in both larval development and egg production in insects, how nutritional information results in increased JH synthesis by the corpora allata glands still largely unknown. Recent evidence from *D. melanogaster* with mutant insulin receptors has shown that corpora allata *in vitro* have greatly reduced rates of JH synthesis. In wild-type flies, axons that react positively to antibodies to allatropin are abundant in the brain and reach into the CC/CA complex. In mutant flies, however, these are less reactive, suggesting that level of allatotropins are correlated with activity of the insulin signalling pathway (Tu *et al*., 2005). An extracted allatotropic peptide that irreversibly stimulates JH production by corpora allata has been detected in the brain and SEG of prepupal honey bees (Rachinsky, 1996). *Manduca* allatotropin irreversibly stimulates release from CA in feeding stage worker larvae (Rachinsky & Feldlaufer, 2000), but does not overcome a block on the last step in JH synthesis (Rachinsky *et al*., 2000). Commercial monoclonal antibody to *Manduca* allatotropin shows no immunoreactivity with brains of bee larvae (Glasscock *et al*., 2005), a result that is consistent with the apparent lack of a protein in the honey bee genome that has significant homology to it. Interestingly, one enzyme that catalyses an early step in JH III synthesis, HMG coenzyme A reductase, is expressed at much higher levels in 3rd-instar queens than in workers of the same age (Evans & Wheeler, 2000).

We have shown that a honey bee insulin-like peptide (*AmILP-1*) is expressed at very high levels in queen larvae at a time that suggests a link between the insulin signalling pathway and the synthesis and release of juvenile hormone. Expression of protein members of the insulin pathway appear to be related in functional ways to diet quality. With the availability of the honey bee genome, expression levels of other important proteins in the insulin signalling pathway, as well as other pathways integrated with it, are now more accessible than ever before.

## Experimental procedures

### Honeybees

Colonies of *Apis mellifera ligustica* were established in a single apiary at the Bee Research Laboratory in Beltsville, MD. Two colonies were kept in a queenless state, with larvae added periodically to maintain a normal age range of adult worker bees. These colonies provided a rearing environment in which queens could be raised. Two additional colonies served as larval sources and as an environment for raising worker-destined larvae.

### Manipulation of larvae

Treatment and sampling design (Fig. 1, the transfer scheme, and Fig. 2, the resulting larval diets): Young first-instar larvae (16 h (range 12 h–18 h) past larval hatch) were identified in the two queenright colonies. A fraction of these larvae were transferred into wax queen-rearing cups while the rest were followed in place by marking adjacent parts of their cell frame. After another 24 h, these larvae had spent either 48 h in a worker cell or 24 h in a worker cell followed by 24 in a queen cell. A proportion of the larvae were collected and frozen at −80 °C these larvae were designated W and Q to indicate the environment in which they had spent the previous 24 h. A proportion of the remaining larvae in each treatment were transferred to the alternate cell type, while the rest were maintained in place. After an additional 24 h, a portion of the larvae in each of four rearing developmental regimes (WW, WQ, QW, QQ) were collected were collected and frozen. Remaining WW and QQ larvae were then transferred to the alternate cell type or maintained in place for 24 h prior to freezing, giving six 96-h (4th instar) developmental regimes (WWW, WWQ, WQQ, QQQ, QQW, QWW). Treatment and sampling were carried out twice and yielded at least 20 larvae in each of the total of 12 categories. In addition, 3 pooled samples of grafting aged (16 h) larvae were collected for analysis.

### Identification of putative insulin pathway genes

Putative genes functioning in the insulin signalling pathway were identified by homology to proteins in *Drosophila melanogaster.*Blast searches on the honey bee genome were carried out in blastp, blastn and tblastx modes. For the current study, homologues of insulin-like peptides, insulin receptor, insulin receptor substrate and PTEN were identified. The described insulin pathway members are present in the consensus (GLEAN3) gene set predicted during the Honey Bee Genome Project. These are *AmILP-1* GB17332-PA GLEAN3–03281, *AmILP-2* GB10174-PA GLEAN3–02304, *AmIR-2* GB18331-PA GLEAN3–03658, *AmIRS* GB11037-PA GLEAN3–08920, and *AmPTEN* GB14441-PA GLEAN3–09958. Expression of a second insulin-receptor candidate, *AmIR-B* (GB15492-PA), was not tested. *AmIR-B* has lower homology than *AmIR-2* with the insulin receptor proteins in *Tribolium castaneum*, *Aedes aegypti*, *D. melanogaster* and humans.

### Primers

Primers were designed based on cDNA sequences from the official gene list or on the basis of FGENESH (http://www.softberry.com) predictions of genomic sequences from proposed regions orthologous to known insulin pathway members. Specific primers used for quantitative PCR were: *AmILP-1* (1L CGATAGTCCTGGTCGGTTTG, 1R CAAGCTGAGCATAGCTGCAC), *AmILP-2* (1L TTCCAGAAATGGAGATGGATG, 1R TAGGAGCGCAACTCCTCTGT), *AmIR-2* (L GGGAAGAACATCGTGAAGGA, R CATCACGAGCAGCGTGTACT), *AmIRS* (L CCACCGAAACGTAGCATTTT, R CAAATATAGGCCGAGGTTGC), *AmPTEN* (1L ACAGCAGTGAATGCGATACG, 1R TTGTGGTTTGCCGATGACTA). The reference was Ribosomal Protein S5e (RPS5) (GB11132-PA Glean3–03503) which had the most consistent expression across conditions for the set of potential references tested. RPS5 was primed with F AATTATTTGGTCGCTGGAATTG, R TAACGTCCAGCAGAATGTGGTA.

### Quantitative Real-time PCR analyses

RNA was extracted separately from individual whole bodies of collected larva using Versagenetm RNA Tissue Kit (Gentra Systems, Minneapolis, MN). First-stranded cDNA was generated using oligo-dT priming and Superscript II (Invitrogen, Carlsbad, CA). qPCR was performed using the protocol provided with MasterMix Plus for SYBR® Green I (Eurogentec, San Diego, CA) and an Applied Biosystems 7300 Real-time PCR System. Relative expression was calculated by comparing cycle thresholds (Cts) to those of the reference gene run simultaneously. Relative expression was calculated by raising 2 to the power of the difference in Ct values. Each sample was run in duplicate and 3–8 samples were run for each of the 12 experimental conditions.

### Statistical Analysis

Statistical analysis was carried out using JMP IN® 5.1 (SAS Institute, Cary, NC). For each gene, the entire experimental design was analysed using an anova. Comparisons among means were made with the Student's t.
